# Data in the activities of caspases and the levels of reactive oxygen species and cytochrome c in the •OH-induced fish erythrocytes treated with alanine, citrulline, proline and their combination

**DOI:** 10.1016/j.dib.2016.02.005

**Published:** 2016-02-09

**Authors:** Huatao Li, Weidan Jiang, Yang Liu, Jun Jiang, Yongan Zhang, Pei Wu, Juan Zhao, Xudong Duan, Xiaoqiu Zhou, Lin Feng

**Affiliations:** a Animal Nutrition Institute, Sichuan Agricultural University, Chengdu 611130, Sichuan, China; b Fish Nutrition and Safety Production University Key Laboratory of Sichuan Province, Sichuan Agricultural University, Chengdu 611130, Sichuan, China; c Key Laboratory for Animal Disease-Resistance Nutrition of Ministry of Education, Sichuan Agricultural University, Chengdu 611130, Sichuan, China; d Institute of Hydrobiology, Chinese Academy of Sciences, Wuhan 430072, Hubei, China; e Conservation and Utilization of Fishes resources in the Upper Reaches of the Yangtze River Key Laboratory of Sichuan Province, Neijiang Normal University, Neijiang 641000, Sichuan, China

## Abstract

The present study explored the effects of alanine (Ala), citrulline (Cit), proline (Pro) and their combination (Ala_10_Pro_4_Cit_1_) on the activities of caspases and levels of reactive oxygen species (ROS) and cytochrome c in hydroxyl radicals (•OH)-induced carp erythrocytes. The data displayed that •OH induced the increases in the activities of caspase−3, caspase−8 and caspase−9 and the levels of ROS and cytochrome c in carp erythrocytes. However, Ala, Cit, Pro and Ala_10_Pro_4_Cit_1_ effectively suppressed the •OH-induced increases in the activities of caspase−3, caspase−8 and caspase−9 and the levels of ROS and cytochrome c in carp erythrocytes. Furthermore, the activities of caspase−3, caspase−8 and caspase−9 and the levels of ROS and cytochrome c were gradually decreased with increasing concentrations of Ala, Cit, Pro and Ala_10_Pro_4_Cit_1_ (0.175−1.400 mM) in the •OH-induced carp erythrocytes. These data demonstrated that the 50% inhibitory doses (ID_50_) of Ala_10_Pro_4_Cit_1_ on the activities of caspase−8, caspase−9 and caspase−3 and levels of ROS and cytochrome c were respectively estimated to be the minimum values among amino acids examined so far. The 5% inhibitory doses (ID_5_) of Ala, Cit, Pro and Ala_10_Pro_4_Cit_1_ on the activities of caspase−8, caspase−9 and caspase−3 and levels of ROS and cytochrome c were estimated to be at their physiological concentrations in mammalian. Our research article for further interpretation and discussion from these data in Li et al. (2016) [1].

**Specifications Table**

**Table:** 

Subject area	*Biology*
More specific subject area	*Nutritional Medicine*
Type of data	*Table, figure*
How data was acquired	*The commercial kit, microplate reader, fluorescence spectrophotometer, SPSS 13.0 for Window software*
Data format	*Analyzed*
Experimental factors	*The isolated carp erythrocytes were suspended in physiological carp saline (PCS) (control) and PCS containing0.000,0.175, 0.350, 0.700 or 1.400* *mMof alanine, citrulline, proline and their combination at a1%hematocrit, respectively. All erythrocyte suspensions above were pre-incubated at37* *°Cfor1.5* *h*. *FeSO*_*4*_*/H*_*2*_*O*_*2*_*was then added at a final concentration of40* *μM/20* *μMfor incubation at37* *°Cfor9* *h*, *except in the control.*
Experimental features	*The measurement of the activities of caspase−3, caspase−8 and caspase−9 and the levels of reactive oxygen species and cytochrome c were performed using the commercial kits.*
Data source location	*Chengdu, Sichuan, China*
Data accessibility	*Data are presented in this article*

**Value of the data**

1. The data provide the information of the effect of different concentrations of alanine (Ala), citrulline (Cit), proline (Pro) and their combination (Ala_10_Pro_4_Cit_1_) on the activities of caspases and levels of reactive oxygen species and cytochrome c in hydroxyl radicals-induced carp erythrocytes.
2. The data would be valuable for further studies of oxidative stress and apoptosis in fish erythrocytes.
3. The data support the development of further experiments on the use of Ala, Cit and Pro as oxidative stress and apoptosis inhibitors in fish cells.
4. The data could give a basis for further experiments on revealing the underlying mechanisms of anti-oxidation and anti-apoptosis of glutamine in mammalian.

## 1 Data

**Fig. 1:**
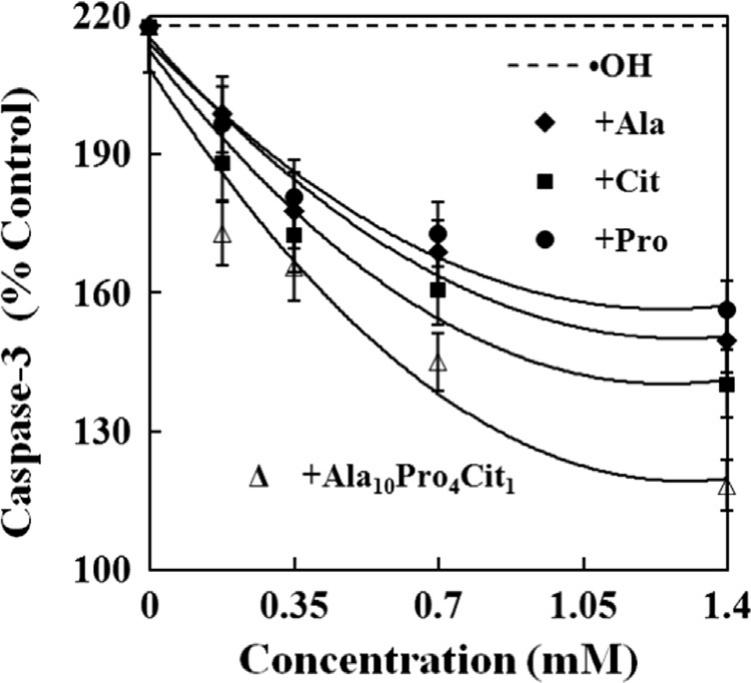
Effects of Ala, Cit, Pro and Ala_10_Pro_4_Cit_1_ on the activity of caspase−3 in the •OH-induced carp erythrocyte. The data represent the mean±S.D. of four replicates.

**Fig. 2:**
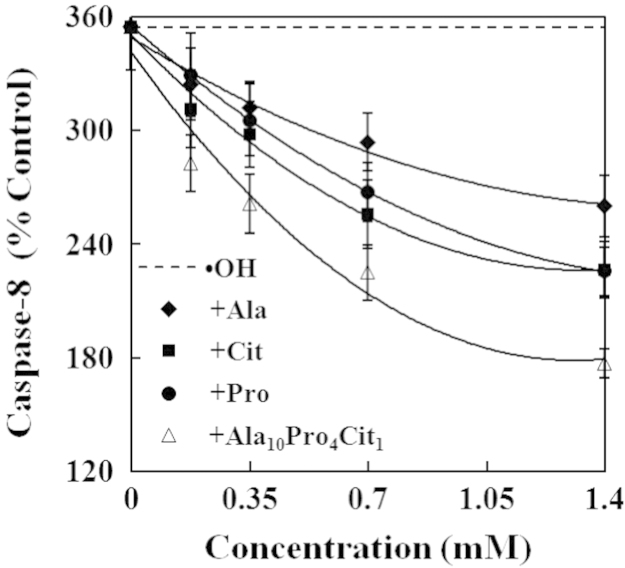
Effects of Ala, Cit, Pro and Ala_10_Pro_4_Cit_1_ on the activity of caspase−8 in the •OH-induced carp erythrocyte. The data represent the mean±S.D. of four replicates.

**Fig. 3:**
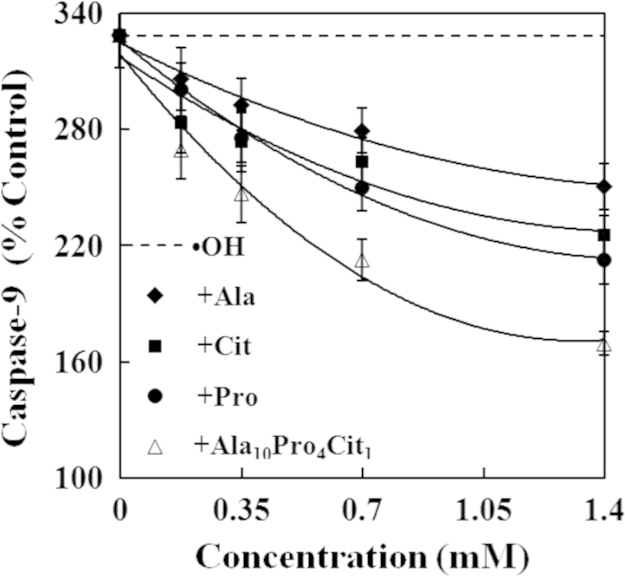
Effects of Ala, Cit, Pro and Ala_10_Pro_4_Cit_1_ on the activity of caspase−9 in the •OH-induced carp erythrocyte. The data represent the mean±S.D. of four replicates.

**Fig. 4:**
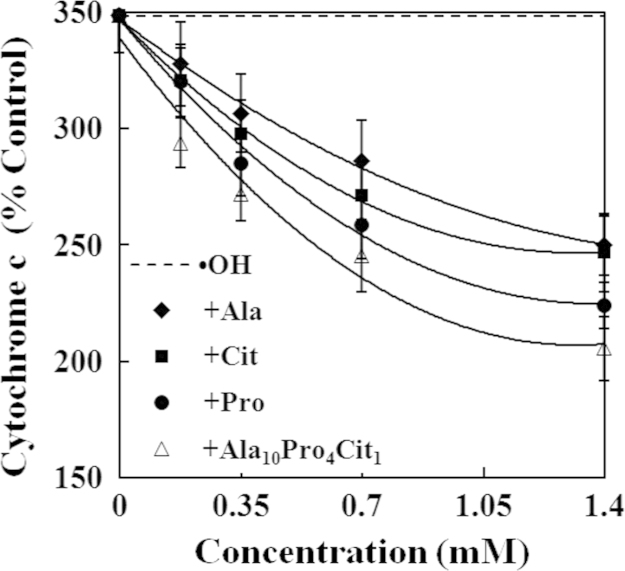
Effects of Ala, Cit, Pro and Ala_10_Pro_4_Cit_1_ on the levels of cytochrome c in the •OH-induced carp erythrocyte. The data represent the mean±S.D. of four replicates.

**Fig. 5:**
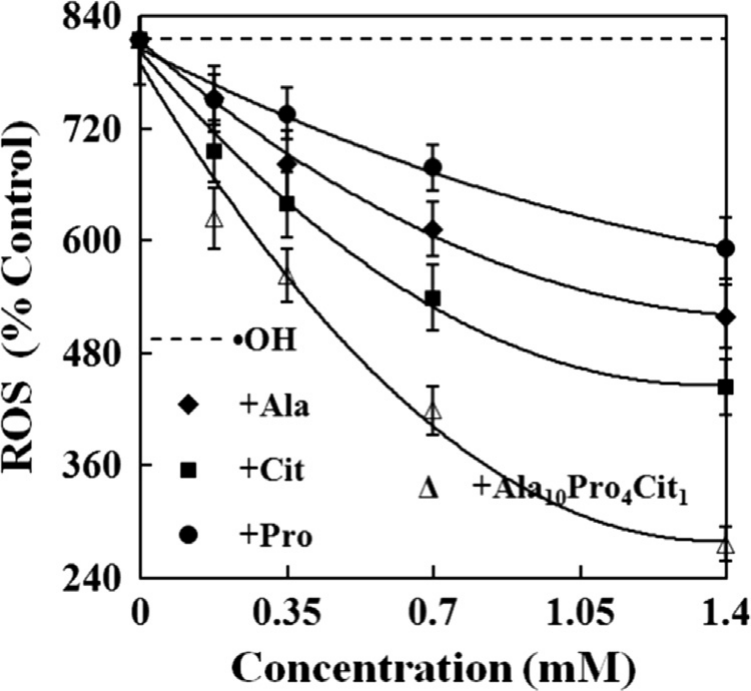
Effects of Ala, Cit, Pro and Ala_10_Pro_4_Cit_1_ on the levels of ROS in the •OH-induced carp erythrocyte. The data represent the mean±S.D. of four replicates.

In the data, the effects of alanine (Ala), citrulline (Cit), proline (Pro) and their combination (Ala_10_Pro_4_Cit_1_) at different concentrations on caspases activities and reactive oxygen species (ROS) and cytochrome c levels in •OH-induced carp erythrocytes were presented in Fig. 1, Fig. 2, Fig. 3, Fig. 4, Fig. 5. Compared with the control, the activities of caspase−3, caspase−8 and caspase−9 and levels of ROS and cytochrome c was significantly increased in carp erythrocytes exposed to •OH. However, Ala, Cit, Pro and Ala_10_Pro_4_Cit_1_ significantly inhibited the •OH-induced increase in the activities of caspases and levels of ROS and cytochrome c in carp erythrocytes. The activities of caspases and levels of ROS and cytochrome c were gradually decreased with increasing concentrations of Ala, Cit, Pro and Ala_10_Pro_4_Cit_1_ (0.175−1.400 mM) in the •OH-induced carp erythrocytes [1].

The data in Table 1 showed that when the erythrocytes were treated with Ala_10_Pro_4_Cit_1_ in the presence of •OH, the 50% inhibitory doses (ID_50_) on the activities of caspase−3, caspase−8 and caspase−9 and levels of ROS and cytochrome c were estimated to be 0.35, 0.60, 0.61, 0.53 and 1.01 mM that are the minimum values among amino acids examined so far [1].

The data in Table 2 showed that the 5% inhibitory doses (ID_5_) of Ala_10_Pro_4_Cit_1_ on the activity of caspase−8 and level of cytochrome c were respectively estimated to be 0.026 and 0.028 mM, the ID_5_ of Ala_10_Pro_4_Cit_1_ and Cit on the activity of caspase−3 or level of ROS were respectively estimated to be 0.024 and 0.025 mM or 0.041 and 0.047 mM, the ID_5_ of Cit on the activity of caspase−9 was estimated to be 0.021 mM, which are the minimum values among amino acids examined so far [1].

## 2 Experimental design, materials and methods

### 2.1 Chemicals

L-Ala, L-Cit and Pro were purchased from Sigma (St. Louis, MO, USA). The aqueous solution of H_2_O_2_ (30%) and FeSO_4_ (analytical pure) were purchased from Shanghai Chemical Reagent Factory (Shanghai, China). All other chemicals were analytical grade. All water used was Milli-Q grade. Physiological carp saline (PCS) (contained 141.1 mM NaCl, 1.43 mM KC1, 0.99 mM CaCl_2_, 2.64 mM NaHCO_3_ and 6.16 mM glucose) modified to give a total osmolarity of 280 mosm L^−1^ and pH 7.9 were prepared in our laboratory.

### 2.2 Erythrocyte isolation

The procedures of cell isolation were based on that described by Phillips et al. (2000) [2] with slight modifications. Individual healthy carp (*Cyprinus carpio* var. Jian) (100–110 g) was anaesthetized, and the blood (approximately 1 ml) was drawn into a syringe via caudal puncture. The blood was placed in a chilled centrifuge tube containing heparinized (40 i.u.ml^−1^) PCS. The blood from three different carp was pooled and centrifugated (Thermo, Waltham, MA, USA) at 900*g* and 4 °C for 10 min. Then, the plasma and buffy coat of pooled blood were removed in the centrifuge tube. After washing three times with PCS, the isolated erythrocytes were resuspended in PCS at a 1% hematocrit in following experiments. All procedures were approved by the Institutional Animal Care and Use Committee of Sichuan Agricultural University in accordance with the Institutional Ethics Committee of the Chinese Institute of Chemical Biology guidelines.

### 2.3 Erythrocyte treatments

Erythrocyte treatments were based on the method described by Li et al. (2013) [3] with slight modifications. Alanine, Cit and Pro were combined in the molar ratio 10:4:1 (Ala_10_Pro_4_Cit_1_) for investigating their mixture effects in the erythrocytes. Briefly, the isolated erythrocytes were suspended in PCS (control) and PCS containing 0.000, 0.175, 0.350, 0.700 or 1.400 mM of Ala, Cit, Pro or Ala_10_Pro_4_Cit_1_ at a 1% hematocrit, respectively. All erythrocyte suspensions above were pre-incubated at 37 °C for 1.5 h. FeSO_4_/H_2_O_2_ was then added at a final concentration of 40 μM/20 μM, except in the control. After incubation at 37 °C for 9 h, the erythrocytes were collected for measurement of the activities of caspase−3, caspase−8 and caspase−9 and the levels of ROS and cytochrome c. The experiment was performed with 4 replicates per treatment and the control.

### 2.4 Measurement of caspases

The activities of caspase−3, caspase−8 and caspase−9 were determined by measuring cleavage of the Ac-DEVD-pNA (caspase−3 substrate), Ac-IETD-pNA (caspase−8 substrate) and Ac-LEHD-pNA (caspase−9 substrate) to pNA using the detection kits (Beyotime, Nantong, China) as described previously [4], [5]. The caspase activity in the samples was quantified with a Microplate reader (Thermo, Waltham, USA) at 405 nm.

### 2.5 Measurement of ROS

ROS levels in the erythrocytes were determined by measuring the oxidative conversion of 2′, 7′-dichlorofluorescin diacetate (DCFH-DA) to fluorescent compound DCF using a detection kit (Beyotime, Nantong, China) as described previously [6], [7], [8]. DCF fluorescence in the erythrocytes was determined at 485 nm excitation and 520 nm emission using a fluorescence spectrophotometer (Thermo, Waltham, USA).

### 2.6 Measurement of cytochrome c

The cytosolic proteins and mitochondria in fish erythrocytes were isolated by a commercial extraction kit (Beyotime, Nantong, China) as described previously [9]. The cytochrome c release from mitochondria in cytosol was evaluated by an enzyme-linked immunosorbent assay (ELISA) kit (ElabScience, Wuhan, China) [10]. Absorbance value in the samples was measured with the Microplate reader at 450 nm.

### 2.7 Protein measurements and statistical analysis

Protein concentration in the hemolysate was measured by Darbkin method [11]. All data are presented as mean±S.D. (*n*≥4). The data were subjected to one-way analysis of variance (ANOVA). If significant differences were found (*P*<0.05), Duncan׳s multiple range tests were used to rank the groups [12]. The 50% and 5% inhibitory doses (ID_50_ and ID_5_) were determined by the probit analysis [3]. All statistical analyses were performed using the SPSS 13.0 for Window software (SPSS, Chicago, IL, USA) [13].

## Figures and Tables

**Table 1:** ID_50_ of Ala, Cit, Pro and Ala_10_Pro_4_Cit_1_ on the activities of caspase−8, caspase−9 and caspase−3 and the levels of cytochrome c and ROS in the • OH-induced carp erythrocytes.

*Treatment*	*Caspase−8 (mM)*	*Caspase−9 (mM)*	*Caspase−3 (mM)*	*Cytochrome c (mM)*	*ROS (mM)*
Ala	3.39±0.24^c^	3.97±0.22^d^	0.95±0.06^c^	2.35±0.16^d^	2.06±0.13^c^
Cit	1.36±0.06^b^	2.52±0.13^c^	0.67±0.04^b^	2.16±0.12^c^	1.28±0.06^b^
Pro	1.37±0.08^b^	1.36±0.07^b^	1.24±0.09^d^	1.33±0.09^b^	4.78±0.23^d^
Ala_10_Pro_4_Cit_1_	0.60±0.04^a^	0.61±0.04^a^	0.35±0.02^a^	1.01±0.06^a^	0.53±0.03^a^

Data represent mean±S.D. of four replicates. The different superscripts "a, b, c, d" indicated that the data are significantly different in the same column base on statistical analysis.

**Table 2:** ID_5_ of Ala, Cit, Pro and Ala_10_Pro_4_Cit_1_ on the activities of caspase−8, caspase−9 and caspase−3 and the levels of cytochrome c and ROS in the •OH-induced carp erythrocytes.

*Treatment*	*Caspase−8 (mM)*	*Caspase−9 (mM)*	*Caspase−3 (mM)*	*Cytochrome c (mM)*	*ROS (mM)*
Ala	0.064±0.004^c^	0.079±0.005^d^	0.044±0.003^c^	0.102±0.006^c^	0.092±0.006^b^
Cit	0.048±0.003^b^	0.021±0.001^a^	0.025±0.002^a^	0.064±0.004^b^	0.047±0.003^a^
Pro	0.100±0.005^d^	0.071±0.004^c^	0.030±0.002^b^	0.068±0.005^b^	0.107±0.008^c^
Ala_10_Pro_4_Cit_1_	0.026±0.002^a^	0.032±0.002^b^	0.024±0.001^a^	0.028±0.002^a^	0.041±0.003^a^

Data represent means±S.D. of four replicates. Values within the same column with different superscripts are significantly different (*p*<0.05).
